# Bone Mineral Density after Weight Gain in 160 Patients with Anorexia Nervosa

**DOI:** 10.3389/fnut.2017.00046

**Published:** 2017-09-29

**Authors:** Najate Achamrah, Moïse Coëffier, Pierre Jésus, Jocelyne Charles, Agnès Rimbert, Pierre Déchelotte, Sébastien Grigioni

**Affiliations:** ^1^Nutrition Unit, Rouen University Hospital, Rouen, France; ^2^Normandie University, UR, INSERM U1073, Rouen, France; ^3^Institute for Research and Innovation in Biomedicine, Rouen, France

**Keywords:** anorexia nervosa, bone mineral density, body composition, fat mass, osteoporosis

## Abstract

Low bone mineral density (BMD) is a frequent complication in anorexia nervosa (AN). There are controversial points of views regarding the restoration of bone mineralization after recovery in AN. We aimed to assess changes of BMD at 3 years in patients with AN and to explore the relationships between body composition, physical activity, and BMD. Patients with AN were included from 2009 to 2011 in a first visit (T0) with evaluation of weight, height, body mass index (BMI), body composition [fat mass (FM) and fat-free mass], and BMD. Those who had low BMD, either osteoporosis or osteopenia, were admitted in a second visit (T1) to carry out a new bone densitometry examination and body composition; they were also asked for their physical activity. At T0, our study involved 160 patients. Low BMD was observed in 53.6% of them and significant factors associated with demineralization were lower BMIs (16.5 ± 2.1 vs 17.3 ± 2.3 kg/m^2^, *p* = 0.01) and higher duration of AN (11.4 ± 10.5 vs 6.4 ± 6.5 years, *p* = 0.001). At 3 years follow-up (T1), 42 patients were involved and no significant changes in BMD were observed despite body weight increase (3.8 ± 6.1 kg). Interestingly, FM gain was a significant factor associated with BMD improvement at follow-up (8.0 ± 9.1 vs 3.0 ± 3.5 kg, *p* = 0.02). Our findings suggest that the restoration of normal bone values is not related to the increase of body weight, at least after 3 years. FM seems to play an important role in the pathophysiological mechanism of osteoporosis and osteopenia in AN.

## Introduction

Anorexia nervosa (AN) is characterized by a difficulty to maintain minimal weight, a fear of gaining weight, a disturbed body image, often associated with denial based on criteria from the Diagnostic and Statistical Manual of Mental Disorders (1). Two subtypes have been descripted, the restricting subtype with primarily loss of weight through significant reductions in caloric intake, and the binge-eating subtype with recurrent binge-eating and purging through self-induced vomiting or laxative misuse. AN is a potentially serious disease; a mortality rate of 5–10% at 10 years has been reported, making it the psychiatric disorder with the highest mortality (2), although more recent studies report less severe mortality rates (3). AN is associated with multiple and severe somatic complications related to malnutrition including bradycardia, hypotension, anemia, and hormonal imbalance (4). AN is also associated with significant psychiatric comorbid conditions, including anxiety, depression, obsessive–compulsive disorders, and excess physical exercise referred as hyperactivity (5, 6).

Low bone mineral density (BMD) is a frequent complication in AN: 38% of patients with AN suffer from osteoporosis and 92% from osteopenia (7). Impaired bone metabolism in AN is multifactorial and includes hormonal changes (8) and reduction of anabolic effect of muscle contraction on the bone, which is directly related to lean body mass (9). Conventional bed rest imposed to patients with AN is probably detrimental on BMD, since immobilization is a well-established risk factor for bone loss (10). Historically, the main treatment of low BMD during AN relies mainly on compensation of calcium-vitamin D depletion (11), but has a limited efficacy in the absence of efficient refeeding (12). Furthermore, the prevalence of vitamin D deficiency among adolescent girls with AN have been reported to be only 2% compared with 24% in healthy controls (13) probably due to an increased use of supplements. Moreover, the benefit of bisphosphonates for the treatment of BMD during AN is not established (14), although Miller et al. have previously reported a significant increase in BMD in adult women with AN with risedronate compared with placebo in a 1-year follow-up study (15). Other studies have reported a decrease in bone turn-over with bisphosphonates, particularly in adolescent patients with AN (16).

Thus, recovery of BMD in AN is described as a slow process with complex interactions between hormonal and nutritional factors (17). There are controversial point of views in the literature regarding the restoration of bone mineralization after recovery in AN. The purpose of this study was to assess changes of BMD at 3 years in patients with AN and to explore the relationships between body composition, physical activity, and BMD at the first visit and at follow-up.

## Materials and Methods

### Population and Study Design

The study started in 2009 with the inclusion of patients with AN diagnosed according to the Diagnostic and Statistical Manual of Mental Disorders-IV criteria. Both restricting subtype (AN-R) and binge-eating subtype (AN-M) were included. This retrospective study involved adult women patients (older than 18 years), addressed by their primary care doctor, from 2009 to 2011. In a first visit (T0), every patient has been evaluated for weight, height, body mass index (BMI), body composition [fat mass (FM) and fat-free mass (FFM)], and BMD. At T0, 160 patients with AN were involved (Table 1). The mean age was 28.3 ± 10 years, and the mean disease duration before the first visit was 7.5 ± 8.3 years. Of the 160 patients, 97 (61.8%) had restricting subtype (AN-R), while 63 (38.2%) had binge-eating subtype (AN-M). Mean weight and BMI was, respectively, 42.2 ± 5.3 kg and 17.06 ± 2.6 kg/m^2^. Mean FM and FFM was, respectively, 19.7 ± 20.2 and 93.5 ± 19.4%.

**Table 1:** Anthropometric data at the first visit (T0).

	Anorexia nervosa (AN) (*n* = 160)	AN-R (*n* = 97; 61.8%)	AN-M (*n* = 63; 38.2%)	*p*
Age (years)	28.3 ± 10	29.1 ± 11.1	27.1 ± 10.4	0.26
Weight (kg)	42.2 ± 5.3	42 ± 5.6	42.7 ± 4.4	0.32
Body mass index (kg/m^2^)	17.4 ± 2.6	16.5 ± 1.9	18.8 ± 3.0	0.01
Mean disease duration (years)	7.5 ± 8.3	6.8 ± 7.5	8.4 ± 9.2	0.12

**Figure 1:**
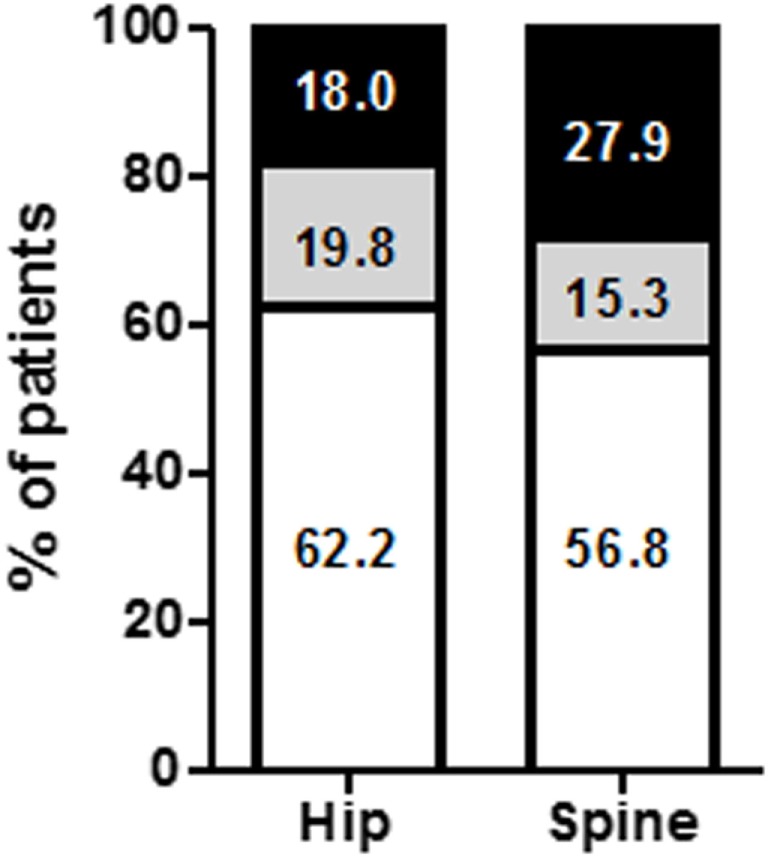
Percentage of anorectic patients according to bone mineral density (BMD) status changes. BMD status was followed in 42 anorexia nervosa (AN) patients. BMD status was classified as BMD loss (open bars), stable (gray bars), or improvement (black bars).

The patients were followed-up in the Department of Clinical Nutrition (University Medical Center, Rouen, France). The patients who had low BMD for age at T0, either osteoporosis or osteopenia were admitted in a second visit (T1) to carry out a new bone densitometry examination and body composition. At T1, patients were also asked for their physical activity. (Do you practice regular exercise?) Since many patients refused to participate or were lost, this second visit involved 42 patients with AN, aged from 22 to 45 years (Figure 1). Of the patients, 33 had pure restrictive form (AN-R) and 9 had mixed form (AN-M). Weight and BMIs was, respectively, 46.1 ± 7.7 kg and 19.01 ± 3.1 kg/m^2^. From T0 to T1, body weight was increased (3.8 ± 6.1 kg). At T1, FM gain was significantly higher in restricting subtype than binge-eating subtype (4.5 ± 4.9 vs 0.5 ± 1.6 kg, *p* < 0.05). Restricting subtype had a slight FFM gain, whereas binge-eating subtype had a FFM loss (+0.3 ± 3.2 vs −0.4 ± 2.2 kg, ns) but difference did not reach significance. The mean duration period from T0 to T1 was 3.0 ± 1.4 years (Table 2).

**Table 2:** Influence of body composition (FM and FFM), body mass index (BMI), and disease duration on bone mineral density at T0.

	Low hip BMD		*p*	Low spine BMD		*p*
	Yes	No		Yes	No	
FFM (%)	84.6 (±8.1)	83.1 (±8.1)	0.24	84.9 (±8.5)	82.7 (±7.7)	0.23
FM (%)	15.1 (±8.3)	16.9 (±8.1)	0.25	14.8 (±8.7)	17.3 (±7.7)	0.08
BMI (kg/m^2^)	16.5 (±2.1)	17.3 (±2.3)	0.01	16.8 (±2.7)	17.1 (±1.8)	0.21
Disease duration (years)	11.4 (±10.5)	6.4 (±6.5)	0.001	9.3 (±8.1)	7.7 (±9.1)	0.11

*BMD, bone mineral density; FFM, fat-free mass; FA, fat mass*.

This study complies with the International Declaration of Helsinki and we obtained a written consent from all the patients. The study was approved by the Local Ethics Committee for Non-Interventional Studies (CERNI).

### Measurement of BMD

Lumbar spine and hip BMD, as well as body composition (FFM and FM), were determined by dual-energy X-ray absorptiometry using Lunar Prodigy Advance osteodensitometer (General Electric Healthcare). The results of BMD were expressed as *T*-scores and we considered that patients with −2.5 ≤ *T*-score < −1 had osteopenia and patients with *T*-score < −2.5 had osteoporosis.

### Statistical Analysis

All statistical analyses were performed using SPSS version 10.0 (SPSS Inc., Chicago, IL, USA), and a *p* < 0.05 was considered to indicate statistical significance. Continuous data were presented as the mean ± SD, and categorical data were presented as count and percentage (%). Statistical analyses between AN subtypes and BMD evolution (recovery or aggravation) were performed using the Chi^2^ test for categorical variables comparison and the Mann–Whitney *U* test for continuous variables. The Wilcoxon signed-rank was used for analyses comparing repeated BMD measurements during the follow-up (T0–T1).

## Results

### First Visit (T0)

#### Bone Mineral Density

**Figure 2:**
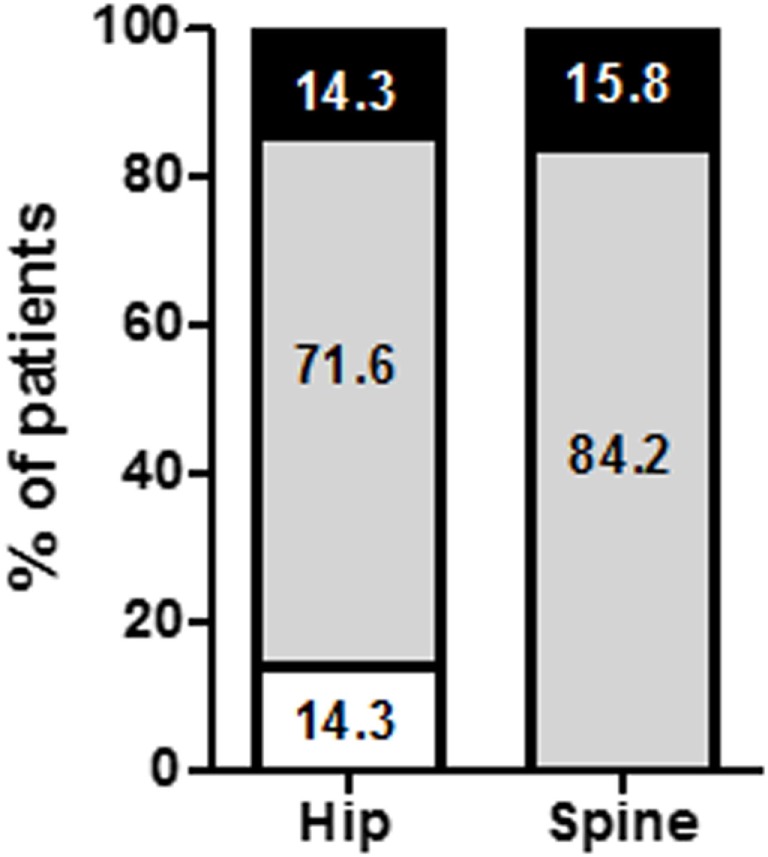
Percentage of anorectic patients according to bone mineral density (BMD) status at inclusion. BMD was evaluated in 160 anorexia nervosa patients who were classified as normal BMD (open bars), osteopenia (gray bars), and osteoporosis (black bars).

At the first visit, low BMD for age (osteoporosis or osteopenia) was observed in 53.6% of the 160 patients (Figure 2). Factors associated with BMD decrease have been identified in Table 2. Patients with low BMD at the hip had significantly lower BMIs (16.5 ± 2.1 vs 17.3 ± 2.3 kg/m^2^, *p* = 0.01) and higher duration of AN (11.4 ± 10.5 vs 6.4 ± 6.5 years, *p* = 0.001). Patients with low BMD at the spine exhibited a trend for a lower FM (14.8 ± 8.7 vs 17.3 ± 7.7%, *p* = 0.08, Table 2).

### Follow-up (T1)

#### Bone Mineral Density

**Figure 3:**
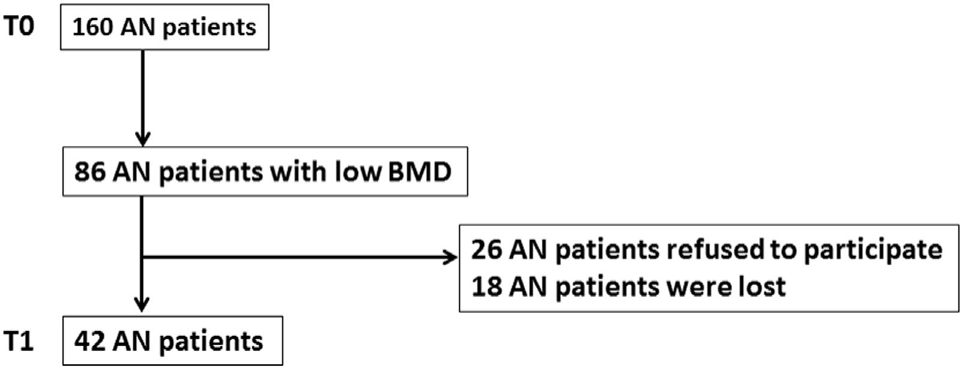
Flow chart. At T0, 160 patients with anorexia nervosa (AN) were involved in the study. Low bone mineral density (osteoporosis or osteopenia) was observed in 86 of those 160 patients. Since 26 patients refused to participate and 18 patients were lost, the second visit (T1) involved 42 patients with AN.

At T1 (Table 3), no significant changes in BMD were observed at the spine or hip (*T*-score: +0.1 ± 0.6 and −0.1 ± 0.5, Table 4). Indeed, BMD remained similar in 71.3 and 84.2% of patients at the hip and spine, respectively (Figure 2). Interestingly, 14.3% of patients had a BMD loss at the hip (Figure 3). We did not find any difference between the AN subtypes for the evolution of BMD (*data no shown*). FM gain was a significant factor associated with BMD improvement at follow-up (8.0 ± 9.1 vs 3.0 ± 3.5 kg, *p* = 0.02, Table 5). We also observed trends for FFM gain (2.4 ± 2.9 vs −0.1 ± 2.9 kg, *p* = 0.08) and weight gain (7.7 ± 8.2 vs 3.2 ± 5.6 kg, *p* = 0.10) as factors associated with BMD improvement (Table 5).

**Table 3:** Anthropometric data at follow-up (T1).

	Anorexia nervosa (AN) (*n* = 42)	AN-R (*n* = 33)	AN-M (*n* = 9)	*p*
Age (years)	33.7 (±11.7)	34.1 (±12)	32.2 (±11.1)	0.24
Weight (kg)	46.1 (±7.7)	46.4 (±8.2)	44.8 (±5.9)	0.22
Δ Weight T0–T1 (kg)	+3.8 (±6.1)	+4.3 (±6.6)	+2.1 (±3.7)	0.11
Δ Body mass index T0–T1 (kg/m^2^)	+1.61 (±3.1)	+0.6 (±3.3)	+0.1 (±2.5)	0.13
Δ T0–T1 (years)	3.0 (±1.4)	3.2 (±1.5)	2.2 (±1.09)	0.01
Δ Fat mass T0–T1 (kg)	+3.7 (4.7)	+4.5 (4.9)	+0.5 (1.6)	0.001
Δ Fat-free mass T0–T1 (kg)	+0.2 (3)	+0.3 (3.2)	−0.4 (2.2)	0.18

**Table 4:** Changes in bone mineral density from T0 to T1.

	T0	T1	Δ T0–T1	*p*
Spine *T*-score	−1.4 (±1.1)	−1.3 (±1.2)	+0.1 (±0.6)	0.21
Hip *T*-score	−1.4 (±1)	−1.6 (±0.9)	−0.1 (±0.5)	0.19

**Table 5:** Influence of body composition [fat mass (FM) and fat-free mass (FFM)] and weight on bone mineral density (BMD) changes.

	BMD improvement		*p*
	Yes	No	
Δ FM T0–T1 (kg)	+8 (±9.1)	+3 (3.5)	0.02
Δ FFM T0–T1 (kg)	+2.4 (2.9)	−0.1 (2.9)	0.08
Δ Weight T0–T1 (kg)	+7.7 (8.2)	+3.2 (5.6)	0.10

	**BMD loss aggravation**		***p***
	**Yes**	**No**

Δ FM T0–T1 (kg)	+3.6 (4.2)	+3.8 (5)	0.36
Δ FFM T0–T1 (kg)	−1 (2.5)	+0.5 (3.1)	0.12
Δ Weight T0–T1 (kg)	2.8 (5.8)	4.2 (6.3)	0.16

#### Physical Activity

Of the 42 patients, 15 patients had a regular physical activity (11 patients AN-R and 4 patients AN-M). Patients exercising had higher FFM gain than inactive patients (+1.3 ± 3.3 vs −0.8 ± 3 kg, *p* = 0.06), whereas FM and body weight were not significantly different. Furthermore, physical activity did not appear as a predictive factor of BMD improvement.

## Discussion

In this study, at the first visit, more than 50% of the population exhibited low BMD either osteopenia or osteoporosis. This result is in accordance with previous studies and confirm the major impact of AN on bone health (18, 19). Moreover, we found a positive correlation between hip BMD and duration of AN as reported in the previous cross-sectional studies (20–22). However, there is emerging evidence that loss of BMD is rapid, occurring relatively early in the disease, even in adolescents with a brief duration of illness. Legroux-Gérot et al. found that spine bone loss correlated negatively with durations of AN in a 1-year follow-up study (23), this finding could be explained by a very rapid process of bone loss at the onset of the disease. In our study, hip BMD was also correlated positively with BMI. This finding is comparable with those reported in the literature (21).

At 3 years follow-up, despite the increase in BMI, bone condition did not significantly change and we did not find any difference between the AN subtypes. Thus, weight recovery did not influence the reestablishment of concrete bone mineralization in our population. Many contradictory results are reported in the literature regarding the reestablishment of bone mineralization after recovery in AN. Some trials have found that weight recovery was related to BMD improvement (18, 24, 25), whereas other recent studies have reported that bone recovery was not influenced by BMI (26, 27). Potential mechanisms involved in the lack of bone recovery after weight restoration are not well understood. Physical hyperactivity has been associated with low bone mass in AN (28). Moreover, increased marrow adiposity, which correlates with low bone density, has been observed in AN (29).

Moreover, we attempted to determine whether or not associations existed between change in BMD at 3 years follow-up and body composition changes. We found a significant positive association between BMD improvement and FM. Indeed, in our population, increase in BMI was mainly related to an increase in FM as previously reported in AN (26). The pathophysiological mechanisms between weight, fat tissue, and bone tissue are increasingly better understood. Leptin is an anorexigenic adipokine mainly produced by fat tissue and has effects on bone. Central leptin is deleterious to the axial skeleton (30, 31) but peripheral leptin has bone anabolic effects with possible osteoclast inhibitory effects (32). AN is often associated with low leptinemia (33), probably an adaptive mechanism to increase appetite, and lower leptin levels are associated with lower FM and bone density measures (34). Furthermore, in a recent study, hypoleptinemia persisted despite the full recovery of FM in an animal model of anorexia (35). Moreover, recent findings are in accordance with our results showing that increasing FM reversed bone loss osteopenia (36).

Lower lean mass is also an important determinant of lower bone density and impaired bone structure in adults and adolescents with AN (37). Previously, Soyka et al. have shown that increases in lean mass following weight gain are strongly predictive of coincident increases in bone density in adolescents with AN (9). In our study, FFM gain was not a significant predictive factor of BMD improvement, even if a trend was observed (2.4 ± 2.9 vs −0.1 ± 2.9 kg, ns). Interestingly, patients exercising had higher FFM gain than inactive patients (+1.3 ± 3.3 vs −0.8 ± 3 kg, *p* = 0.06). A few studies have suggested that moderate exercise may be protective against osteoporosis in women with AN, whereas pathological hyperactivity may be harmful (38, 39). It is generally accepted that exercise help in maintaining BMD in postmenopausal women and increase BMD of the spine and hip in women with osteopenia and osteoporosis (40). Therefore, some authors have hypothesized that, through increasing lean body mass, physical activity may improve BMD during AN management to compensate in part for the hypogonadism (28). However, in our study, physical activity did not appear as a predictive factor of BMD improvement, maybe because of the small population. Thus, prospective and controlled studies are needed to better understand how adapted physical activity may influence BMD evolution during the treatment of AN and may be integrated in a multimodal preventive or curative treatment of low BMD.

Because of its retrospective nature, the main limit of this study is the lake of correlation with biological parameters such as leptinemia or 25-OH vitamin D levels. Moreover, we did not provide detail on either patient menstrual status or type and intensity of exercise. However, this trial has some strength: the long-term follow-up, the evaluation of bone, body composition, and impact of physical activity which have been poorly studied in AN.

In conclusion, our findings suggest that the reestablishment of normal bone values is not related to the increase in body weight, at least after 3 years. Duration of AN, FM, hormonal factors seem to play an important role in the pathophysiological mechanism of osteoporosis and osteopenia in AN. Further trials are needed to better understand this complex pathways in AN.

## Ethics Statement

This project was approved by the “Local Ethics Committee for Non-Interventional studies” (CERNI, Comité d’Ethique local pour la Recherche Non Interventionnelle). This study complies with the International Declaration of Helsinki and we obtained a written consent from all the patients.

## Author Contributions

NA, MC, and SG designed the study. NA, PJ, AR, PD, and SG included patients. MC and JC performed BMD and body composition acquisition. NA and SG performed statistical analysis. NA wrote the first draft of the manuscript. All the authors performed critical revision of the manuscript and approved the final version of the manuscript.

## Conflict of Interest Statement

The authors declare that the research was conducted in the absence of any commercial or financial relationships that could be construed as a potential conflict of interest.

## References

[1] Battle DE Diagnostic and statistical manual of mental disorders (DSM). Codas (2013) 25:191–2. 10.1590/S2317-17822013000200017 24413388

[2] Harris EC Barraclough B. Excess mortality of mental disorder. Br J Psychiatry (1998) 173:11–53. 10.1192/bjp.173.1.11 9850203

[3] Rigaud D Pennacchio H Bizeul C Reveillard V Vergès B. Outcome in AN adult patients: a 13-year follow-up in 484 patients. Diabetes Metab (2011) 37:305–11. 10.1016/j.diabet.2010.11.020 21317006

[4] Miller KK Grinspoon SK Ciampa J Hier J Herzog D Klibanski A. Medical findings in outpatients with anorexia nervosa. Arch Intern Med (2005) 165:561–6. 10.1001/archinte.165.5.561 15767533

[5] Anderluh MB Tchanturia K Rabe-Hesketh S Treasure J. Childhood obsessive-compulsive personality traits in adult women with eating disorders: defining a broader eating disorder phenotype. Am J Psychiatry (2003) 160:242–7. 10.1176/appi.ajp.160.2.242 12562569

[6] Godart NT Flament MF Lecrubier Y Jeammet P. Anxiety disorders in anorexia nervosa and bulimia nervosa: co-morbidity and chronology of appearance. Eur Psychiatry (2000) 15:38–45. 10.1016/S0924-9338(00)00212-1 10713801

[7] Mitchell JE Crow S. Medical complications of anorexia nervosa and bulimia nervosa. Curr Opin Psychiatry (2006) 19:438–43. 10.1097/01.yco.0000228768.79097.3e 16721178

[8] Legroux-Gérot I Vignau J Biver E Pigny P Collier F Marchandise X Anorexia nervosa, osteoporosis and circulating leptin: the missing link. Osteoporos Int (2010) 21:1715–22. 10.1007/s00198-009-1120-x 20052458

[9] Soyka LA Misra M Frenchman A Miller KK Grinspoon S Schoenfeld DA Abnormal bone mineral accrual in adolescent girls with anorexia nervosa. J Clin Endocrinol Metab (2002) 87:4177–85. 10.1210/jc.2001-011889 12213868

[10] Ivuskans A Maestu J Jurimae T Lätt E Purge P Saar M Sedentary time has a negative influence on bone mineral parameters in peripubertal boys: a 1-year prospective study. J Bone Miner Metab (2015) 33(1):85–92. 10.1007/s00774-013-0556-4 24549738

[11] Kitchin B Morgan SL. Not just calcium and vitamin D: other nutritional considerations in osteoporosis. Curr Rheumatol Rep (2007) 9:85–92. 10.1007/s11926-007-0027-9 17437673

[12] Dede AD Lyritis GP Tournis S Bone disease in anorexia nervosa. Hormones (Athens) (2014) 13:38–56. 24722126 10.1007/BF03401319

[13] Haagensen AL Feldman HA Ringelheim J Gordon CM. Low prevalence of vitamin D deficiency among adolescents with anorexia nervosa. Osteoporos Int (2008) 19:289–94. 10.1007/s00198-007-0476-z 17924053 PMC3199303

[14] Misra M Klibanski A. Anorexia nervosa and bone. J Endocrinol (2014) 221:R163–76. 10.1530/JOE-14-0039 24898127 PMC4047520

[15] Miller KK Meenaghan E Lawson EA Misra M Gleysteen S Schoenfeld D Effects of risedronate and low-dose transdermal testosterone on bone mineral density in women with anorexia nervosa: a randomized, placebo-controlled study. J Clin Endocrinol Metab (2011) 96:2081–8. 10.1210/jc.2011-0380 21525157 PMC3135194

[16] Misra M Miller KK Bjornson J Hackman A Aggarwal A Chung J Alterations in growth hormone secretory dynamics in adolescent girls with anorexia nervosa and effects on bone metabolism. J Clin Endocrinol Metab (2003) 88:5615–23. 10.1210/jc.2003-030532 14671143

[17] Valla A Groenning IL Syversen U Hoeiseth A. Anorexia nervosa: slow regain of bone mass. Osteoporos Int (2000) 11:141–5. 10.1007/PL00004175 10793872

[18] Olmos JM Valero C del Barrio AG Amado JA Hernández JL Menéndez-Arango J Time course of bone loss in patients with anorexia nervosa. Int J Eat Disord (2010) 43:537–42. 10.1002/eat.20731 19658172

[19] Soyka LA Grinspoon S Levitsky LL Herzog DB Klibanski A. The effects of anorexia nervosa on bone metabolism in female adolescents. J Clin Endocrinol Metab (1999) 84:4489–96. 10.1210/jc.84.12.4489 10599707

[20] Miller KK Lee EE Lawson EA Misra M Minihan J Grinspoon SK Determinants of skeletal loss and recovery in anorexia nervosa. J Clin Endocrinol Metab (2006) 91:2931–7. 10.1210/jc.2005-2818 16735492 PMC3220933

[21] Legroux-Gérot I Vignau J D’Herbomez M Collier F Marchandise X Duquesnoy B Evaluation of bone loss and its mechanisms in anorexia nervosa. Calcif Tissue Int (2007) 81:174–82. 10.1007/s00223-007-9038-9 17668143

[22] Bachrach LK Guido D Katzman D Litt IF Marcus R. Decreased bone density in adolescent girls with anorexia nervosa. Pediatrics (1990) 86:440–7. 2388792

[23] Legroux-Gérot I Vignau J D’Herbomez M Flipo RM Cortet B. Predictive factors of change in BMD at 1 and 2 years in women with anorexia nervosa: a study of 146 cases. Osteoporos Int (2012) 23:2855–61. 10.1007/s00198-012-1919-8 22349911

[24] Misra M Prabhakaran R Miller KK Goldstein MA Mickley D Clauss L Weight gain and restoration of menses as predictors of bone mineral density change in adolescent girls with anorexia nervosa-1. J Clin Endocrinol Metab (2008) 93:1231–7. 10.1210/jc.2007-1434 18089702 PMC2291495

[25] El Ghoch M Gatti D Calugi S Viapiana O Bazzani PV Dalle Grave R. The association between weight gain/restoration and bone mineral density in adolescents with anorexia nervosa: a systematic review. Nutrients (2016) 8:E769. 10.3390/nu8120769 27916839 PMC5188424

[26] Franzoni E Ciccarese F Di Pietro E Facchini G Moscano F Iero L Follow-up of bone mineral density and body composition in adolescents with restrictive anorexia nervosa: role of dual-energy X-ray absorptiometry. Eur J Clin Nutr (2014) 68:247–52. 10.1038/ejcn.2013.254 24346474

[27] Halvorsen I Platou D Hoiseth A. Bone mass eight years after treatment for adolescent-onset anorexia nervosa. Eur Eat Disord Rev (2012) 20:386–92. 10.1002/erv.2179 22552854

[28] Achamrah N Coeffier M Dechelotte P. Physical activity in patients with anorexia nervosa. Nutr Rev (2016) 74:301–11. 10.1093/nutrit/nuw001 27052638

[29] Greco EA Lenzi A Migliaccio S. The pathophysiological basis of bone tissue alterations associated with eating disorders. Horm Mol Biol Clin Investig (2016) 28:121–32. 10.1515/hmbci-2016-0006 26985689

[30] Ducy P Amling M Takeda S Priemel M Schilling AF Beil FT Leptin inhibits bone formation through a hypothalamic relay: a central control of bone mass. Cell (2000) 100:197–207. 10.1016/S0092-8674(00)81558-5 10660043

[31] Hamrick MW Pennington C Newton D Xie D Isales C. Leptin deficiency produces contrasting phenotypes in bones of the limb and spine. Bone (2004) 34:376–83. 10.1016/j.bone.2003.11.020 15003785

[32] Hamrick MW Della-Fera MA Choi YH Pennington C Hartzell D Baile CA. Leptin treatment induces loss of bone marrow adipocytes and increases bone formation in leptin-deficient ob/ob mice. J Bone Miner Res (2005) 20:994–1001. 10.1359/JBMR.050103 15883640

[33] Śmiarowska M Safranow K Dziedziejko V Bialecka M Koziołek M Samochowiec J. Association of plasma hormones, nutritional status, and stressful life events in anorexia nervosa patients. Postepy Hig Med Dosw (Online) (2014) 68:162–71. 10.5604/17322693.1088743 24662784

[34] Lawson EA Miller KK Bredella MA Phan C Misra M Meenaghan E Hormone predictors of abnormal bone microarchitecture in women with anorexia nervosa. Bone (2010) 46:458–63. 10.1016/j.bone.2009.09.005 19747572 PMC2818221

[35] Zgheib S Méquinion M Lucas S Leterme D Ghali O Tolle V Long-term physiological alterations and recovery in a mouse model of separation associated with time-restricted feeding: a tool to study anorexia nervosa related consequences. PLoS One (2014) 9:e103775. 10.1371/journal.pone.0103775 25090643 PMC4121212

[36] Hedges WP Bukhari M. Increasing body fat mass reverses bone loss in osteopenia as detected by dual-energy X-ray absorptiometry scans. Eur J Rheumatol (2016) 3:1–4. 10.5152/eurjrheum.2015.0025 27708960 PMC5042266

[37] Faje AT Karim L Taylor A Lee H Miller KK Mendes N Adolescent girls with anorexia nervosa have impaired cortical and trabecular microarchitecture and lower estimated bone strength at the distal radius. J Clin Endocrinol Metab (2013) 98:1923–9. 10.1210/jc.2012-4153 23509107 PMC3644600

[38] Joyce JM Warren DL Humphries LL Smith AJ Coon JS. Osteoporosis in women with eating disorders: comparison of physical parameters, exercise, and menstrual status with SPA and DPA evaluation. J Nucl Med (1990) 31:325–31. 2308003

[39] Rigotti NA Nussbaum SR Herzog DB Neer RM. Osteoporosis in women with anorexia nervosa. N Engl J Med (1984) 311:1601–6. 10.1056/NEJM198412203112503 6504095

[40] Zehnacker CH Bemis-Dougherty A. Effect of weighted exercises on bone mineral density in post menopausal women. A systematic review. J Geriatr Phys Ther (2007) 30:79–88. 10.1519/00139143-200708000-00007 18171491

